# Use of Cumulative Live Birth Rate per Total Number of Embryos to Calculate the Success of IVF in Consecutive IVF Cycles in Women Aged ≥35 Years

**DOI:** 10.1155/2019/6159793

**Published:** 2019-06-26

**Authors:** Meng Zhang, Tao Bu, Haiqing Tian, Xia Li, Duolao Wang, Xiaohui Wan, Qingli Wang, Xinmin Mao, Xiaolin La

**Affiliations:** ^1^Xinjiang Medical University, the First Affiliated Hospital, Urumqi, Xinjiang, China; ^2^Liverpool School of Tropical Medicine, Liverpool L3 5QA, UK

## Abstract

**Objective:**

The use of cumulative live birth rate (CLBR) per ovarian stimulation cycle is proving to be an accurate method to calculate the success of IVF; however, the cycle outcome is closely associated with the number of embryos transferred (ET). Our aim was to report CLBR after IVF according to the number of embryos required to achieve a live birth in women aged ≥35 years, considering age, body mass index (BMI), and ethnicity.

**Methods:**

We conducted a retrospective cohort study including 1344 patients who underwent IVF between January 2013 and June 2016 at the First Affiliated Hospital of Xinjiang Medical University. The cumulative probability of live birth for each couple was estimated using the Kaplan–Meier method, and survival curves were compared according to age, BMI, and ethnicity using the log-rank test.

**Results:**

CLBR increased rapidly from 1 to 5 ETs, moderately from 6 to 10 ETs, and slowly thereafter. CLBR was significantly different across 4 categories based on BMI as well as across those based on age; low CLBR was significantly associated with the age of ≥42 years and obesity.

**Conclusion:**

The association between CLBR and number of ET provides realistic and precise information regarding the success of IVF and can be applied to guide clinicians and patients.

## 1. Introduction

The emergence of assisted reproductive technology (ART) has brought hope to infertile couples. Since the birth of the world's first “test tube baby,” Louise Brown, 40 years ago, 8 million babies have been conceived worldwide through ART [1]. However, IVF success rate remains far below 100%. Therefore, the ratio of probability of success and cost effectiveness is an important indicator of decision making and should be considered as a key point for patients who have a low ratio [2].

Typically, cumulative live birth rate (CLBR) and pregnancy rate are used as key indicators to assess the quality and success of IVF, and these are indispensable for patients to make decisions before starting the treatment [3, 4]. CLBR allows for relevant comparison between different reproductive medical centers. Moreover, it may help patients make decisions regarding continuing treatment or remaining childless.

There is a growing tendency in some countries for couples to have children late in their life. This situation is obvious in China for several reasons, such as the two-child policy issued in 2015 [5]. In addition, Chinese women tend to receive education for a prolonged period during their lifespan, which means that they may get married late and consequently postpone childbearing; women with higher education may focus more attention on their career. Apart from these reasons involving women, the high cost of living (burden of feeding a family and offering high-quality education for children) may contribute to late marriages.

According to a recent study, nearly 57% of the couples of all ages in United Kingdom remained childless even after undergoing 6 complete IVF cycles [6]. Female age is the main factor related to delivering a child through ART [7]. That is, women aged ≥35 years would show a lower live birth rate in a single cycle than younger women. Thus, patients older than 35 years of age require multiple cycles to increase the probability of live birth. However, many patients discontinue treatment after 1 or 2 failed treatment cycles at the beginning of ART, although the final prognosis of achieving a live birth may remain promising. Furthermore, the number of embryos transferred (ET) in each cycle varies from 1 to 3, with 3 being the maximum. This reinforces that CLBR per cycle may not be a reliable index for providing informed advice to patients.

The aim of this study was to calculate CLBR per total number of ET required for achieving a live birth. This pragmatic method predicted outcomes considering each embryo as single opportunity to achieve a live birth. This approach is advantageous in that it can provide doctors and patients with a more accurate probability of live birth. Female age, BMI, and ethnicity were considered the key factors affecting the success of IVF.

## 2. Methods

### 2.1. Study Design and Samples

We performed a retrospective cohort study including 1344 women aged 35–45 years who underwent IVF/ET between January 2013 and June 2016 at the Reproductive Medicine Center, the First Affiliated Hospital of Xinjiang Medical University, Xinjiang, China. The inclusion criteria were as follows: (1) women with a ≥1-year history of infertility and age of ≥35 years; (2) those undergoing the first cycle of IVF/ICSI; and (3) those in whom own oocytes were used and fresh or frozen-thawed embryos were transferred without preimplantation genetic screening for embryo aneuploidies. The exclusion criteria were as follows: (1) patients undergoing IVF cycles without ET for any reason; (2) those unsuitable for treatment because of severe disease; (3) those with unknown clinical outcome; (4) those aged >45 years; and (5) those with congenital uterine dysplasia. This study was approved by the Institutional Review Board (IRB) of the First Affiliated Hospital of Xinjiang Medical University, and all patients provided written informed consent before treatment.

### 2.2. Follow-Up

All patients were followed up for at least 2 years or until (1) they decided to discontinue the IVF treatment; (2) all embryos were transferred; (3) they decided to change to ovum donation; (4) had delivery of at least 1 live child surviving over 1 month; or (5) they were still receiving IVF treatment at the end of the study.

### 2.3. Data Collection

Data were obtained from the ART records of the Reproductive Medicine Center without any patient identifier information. The following information was collected: maternal age (≤37 years, 38–39 years, 40–41 years, and ≥42 years); ethnicity (Han, Vygur, and others); basal serum FSH level; basal luteinizing hormone level; infertility duration; body mass index (BMI; underweight: <18.5 kg/m^2^, moderate: <18.5–24.9 kg/m^2^, overweight: 25.0–29.9 kg/m^2^, and obese: >30 kg/m^2^); retrieved oocyte number; gonadotropin (Gn) dosage, Gn day, total number of ET (number of ET to achieve a live birth (ETRL); 1–3 ETRL, 4–6 ETRL, 7–9 ETRL, and >10 ETRL); number of metaphase II oocytes obtained; and outcomes in terms of pregnancy (include ongoing pregnancy), miscarriage, and live birth. The primary outcome was live birth, defined as any birth event in which at least 1 baby was born alive and survived for more than 1 month.

### 2.4. Statistical Analysis

To summarize the characteristics of patients, continuous variables were expressed as mean (standard deviation, SD) or median (interquartile range, IQR) and categorical variables were expressed as frequency (percentage). Categorical variables were compared using the* χ*^2^ or Fisher's exact test. Continuous variables were compared using t test, analysis of variance, or Mann–Whitney U test, depending on data distribution.

The cumulative probability of live birth for each couple undergoing IVF/ICSI treatment during the study period was calculated using the Kaplan–Meier method; this was according to the total ET in each cycle of treatments required to achieve a live birth. The probability of cumulative live birth and outcomes were estimated by the Kaplan–Meier method and compared using the log-rank, Breslow, and Tarone–Ware tests according to each categorical group. A two-tailed* P*< 0.05 was considered statistically significant in all analyses. All analyses were performed using Stata 13 and R version 3.3.1 (http://www.r-project.org/).

## 3. Results

### 3.1. Baseline Characteristics

The cohort included 1344 patients aged 35–45 years who underwent IVF/ICSI cycles involving 2596 ET. Demographical and clinical characteristic of patients are summarized in Tables 1 and 2. Mean and median number of embryos transferred was 1.7(SD: 0.48) and 2 (IQR: 1.5-2); mean and median number of embryos transfers was 3.5(SD: 2.28) and 3 (IQR: 2-3). Patients were assigned to 4 groups according to the number of ET: 1–3 ETRL group with 810 patients (60.3%); 4–6 ETRL group with 392 patients (29.2%); 7–9 ETRL group with 111 (8.2%); and >10 ETRL group with 31 patients (2.3%); only 1 patient required 15 ETRL (Supplementary Table 1). During the study period, a total of 549 pregnancies were confirmed, of which 92 pregnancies ended in a miscarriage; therefore, 457 patients achieved a live birth with a CLBR of 34.0% (included 403 singletons and 54 twins). The >10ETRL group showed the highest miscarriage rate and the lowest CLBR. CLBR of the 4 ETRL groups was 34.94%, 32.12%, 37.82%, and 19.35%, respectively.

### 3.2. Overall and Stratified CLBRs

**Figure 1:**
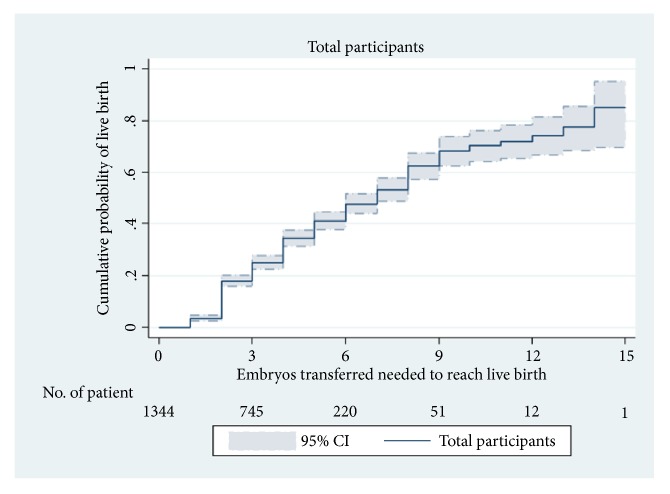
Kaplan–Meier curves for CLBR according to the total number of ET required to achieve a live birth in all participants.

Figure 1 presents the overall CLBR of all participants. After 5 ETRL, the cumulative rate was 41.1% (95% CI: 37.7%–44.7%), representing an approximate increase at an average of 8% per additional embryo. When CLBR reached 70.4% (95% CI: 64.3%–76.2%) after 10ETRL and 85% after 15 ETRL, the increase in cumulative live birth rate was only 3% per embryo.

**Figure 2:**
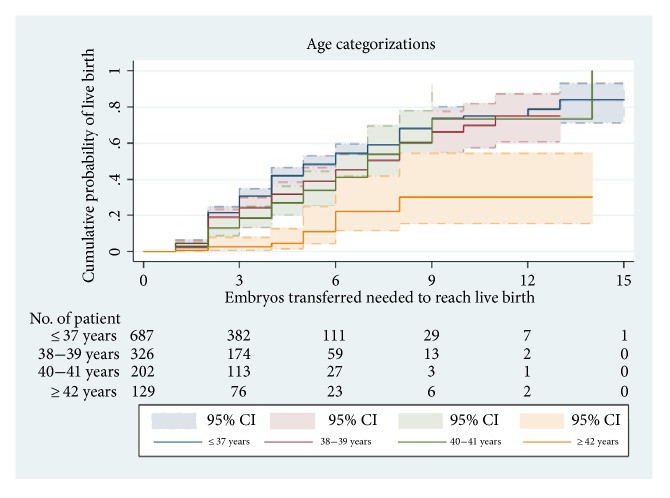
Kaplan–Meier curves for CLBR according to the total number of ET required to achieve a live birth in all age categories.

Figure 2 presents CLBR according to age. Among women aged <42 years, CLBR was approximately 50% after 6 ETRL. Among women aged ≥42 years, there was a dramatic reduction in CLBR to 25%. CLBR was significantly different among the 4 age groups (log-rank test:* χ*^2^ = 44.38, P < 0.0001).

**Figure 3:**
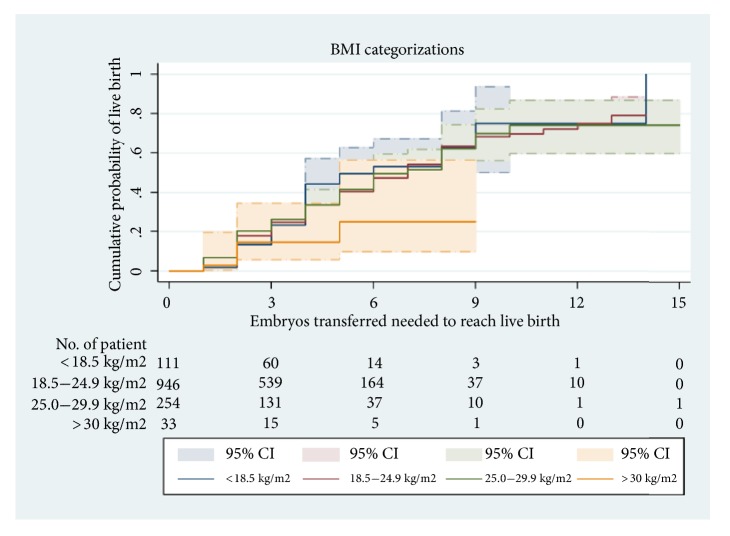
Kaplan–Meier curves for CLBR according to the total number of ET required to achieve a live birth in all BMI categories.

Figure 3 presents CLBR according to BMI. CLBR significantly decreased in patients with BMI of >30 kg/m^2^. After 5 ETRL, CLBR was approximately 50%, 42%, 40%, and 25%, respectively. Obese women showed CLBR limited to 25% after 5 ETRL. CLBR was significantly different across BMI categories (log-rank test:* χ*^2^ = 65.29, P < 0.0001).

**Figure 4:**
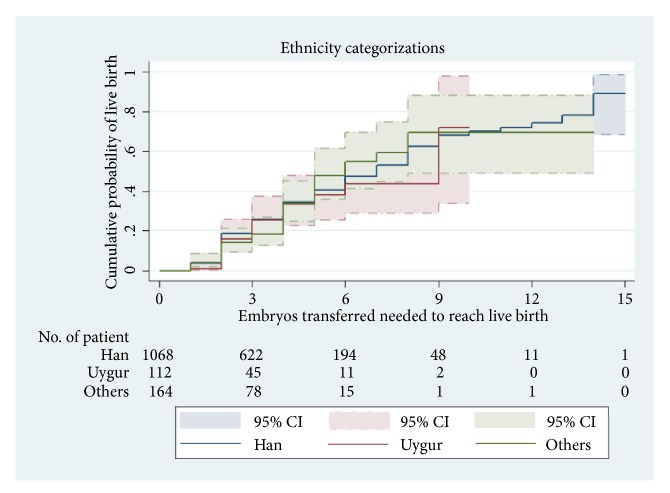
Kaplan–Meier curves for CLBR depending on the total number of ET required to achieve a live birth in all ethnicity categories.

CLBR was not significantly different across ethnicity categories (log-rank test:* χ*^2^ = 0.53, P = 0.7674; Figure 4).

## 4. Discussion

We evaluated outcomes of IVF treatment according to the total number of ET until a term live birth was achieved in women aged ≥35 years. Although the patients underwent the same number of treatment cycles, the total number of ET varied because the number of ET in each cycle varied from 1 to 3. Therefore, considering each embryo as a single opportunity for achieving a live birth can be a useful method to evaluate the outcomes of IVF/ICSI treatment, and it has the advantage of providing more accurate probability of treatment success to clinicians and patients. The cumulative probability of live birth was nearly 25% with 3 ET but only half of this rate with 6 ET. This indicates that the number of ET is a determinant of pregnancy and live birth achievements.

The number of ET was an essential factor associated with CLBR. This is generally in agreement with other studies showing that the number oocytes retrieved was a contributing factor to live birth rate after IVF, with recent evidence indicating that CLBR increases with the number of oocytes retrieved [8–11]. Not all oocytes can completely develop into a mature egg and be successfully fertilized to become an embryo. Therefore, to obtain 5 embryos, approximately 14 metaphase II oocytes are required, with a fertilization rate of 70% such that 50% of them could develop to a blastocyst-stage embryo [12, 13]. Furthermore, patients with polycystic ovary syndrome (PCOS) typically produce more oocytes, but they are often of poor quality and show a low rate of fertilization [14, 15]. Therefore, it seems appropriate and reasonable to evaluate the IVF outcomes by considering the number of ET.

Female age is the main factor to achieve a successful live birth through ART. Natural aging may reduce fertility in a patient seeking IVF treatment [16]. Women aged ≥35 years present increased risk of abortion, significantly reduced pregnancy and live birth rates, and increased risk of pregnancy comorbidities and complications [17–20]. Women aged ≥46 years were excluded from this study because their pregnancy rate tended to be 0% under a natural condition [21], and a recent study suggested that IVF treatment was futile in women aged ≥44 years (CLBR of 3% was never reached irrespective of the number of retrieved oocytes) [11]. Studies indicated that embryo quality is affected by the age of the woman [22–24]. Age was found to be a predictor for the number of retrieved oocytes, number of metaphase II oocytes and embryo quality [25]. Declines in oocytes yields and oocytes quality are major reasons for deteriorating IVF outcomes with advanced female age. Embryo quality was largely determined by oocytes and embryo quality determines most of pregnancy and live birth probability. A possible explanation for this is that women's fertility decreases with increasing age due to a combination of a lowered number of embryos for transfer and decline in embryo quality associated with factors including chromosomal aneuploidy, chromosomal errors, fragmentation, and mitochondrial dysfunction [26–28].

Our results show that IVF overcomes infertility in women aged ≤41 years. Women at ≥42 years should be informed that IVF treatment cannot completely overcome reduced fertility due to age, which is consistent with reports of a previous study [11]. The added value of the present findings is that, for women aged ≥42 years, discontinuation of further treatment may be recommended after 8 embryos are transferred. Moreover, women of advanced age should be informed of the increased risk of pregnancy and perinatal complications. Women who become pregnant at the age of ≥40 years face increased risks of stroke and heart attack later in their life compared with women who become pregnant at a younger age [29]. Increased risk of neonatal birth defects such as Down syndrome and cerebral palsy is associated with increased age during pregnancy [18, 30].

Studies assessing the potential impact of female BMI on IVF outcomes have reported conflicting results. Multiple studies and a meta-analysis indicated that higher BMI was associated with fewer retrieved oocytes, higher cancellation rates, higher miscarriage rate, and lower pregnancy and live birth rates [31–36]. Meanwhile, other studies reported no adverse effect of increased BMI on IVF outcomes [37–40]. This difference in conclusions may be due to disparities of ethnicity, dietary structure, lifestyle, genetics, and environmental factors. Therefore, we categorized BMI into 4 groups and applied the Kaplan–Meier method to analyze CLBR of each group. Obese women showed the lowest CLBR among these 4 groups, and CLBR was limited to 25% after 5 embryos were transferred. Obese women show dysregulated meiotic spindle formation, which may lead to reduced size and quality of oocytes and embryos [41, 42]. Thus, obese women should be counseled regarding the detrimental effects of obesity and benefits of weight loss, including improvements in treatment outcomes, as recommended by a recent meta-analysis.

Our study has several strengths. We considered each embryo as a single opportunity for achieving live birth. The plots we produced are easy for clinicians and patients to understand and show the specific probability of treatment success according to patient characteristics. We did not restrict the treatment to 6 cycles as did other studies, [6] allowing us to assess prognosis to a considerable degree. Nonetheless, our study has several limitations. First, the etiology of infertility was not considered. There are numerous reasons for male and female infertility, and some etiologies, such as mild male factor infertility and low ovarian response, are difficult to define and classify. Second, we did not consider embryo quality owing to the lack of standard quantifiable items to evaluate it. Third, we did not account for confounders such as smoking status and alcohol intake. Finally, the retrospective nature of the study cannot exclude all biases.

## 5. Conclusions

In summary, the present study provides the expected probability of having a child based on the number of ET. Clinicians can provide accurate probabilities to patients using visual graphics such as the Kaplan–Meier curves. This may assist patients to make difficult decisions regarding whether to pursue the IVF/ICSI treatment. Women aged ≥42 years may not experience any benefit with CLBR after 8 embryos are transferred; thus, they should be discouraged from continuing IVF treatment. Obese women seeking fertility treatment should be counseled on the detrimental effects of obesity and benefits of weight reduction, and they should be informed that weight loss via diet and exercise is associated with improved probability of becoming pregnant, with a trend toward improved live birth rate.

## Figures and Tables

**Table 1:** Clinical characteristics of the treatments according to the number of embryos transferred to reach live birth (ETRL).

	From 1–3 ETRL (n=810)	From 4–6 ETRL (n=392)	From 7–9 ETRL (n=111)	More than 10 (n=31)	Overall *P *
Female age (years)	37.91±2.41	37.90±2.45	37.65±2.37	37.55±2.19	0.6300
Male age (years)	39.17±4.31	39.03±4.32	38.79±3.82	39.74±3.66	0.6565
BMI (kg/m2)	23.01±3.09	23.00±2.98	23.12±3.18	23.13±3.03	0.9824
Basal FSH	9.14±3.54	8.33±2.91	7.88±2.69	7.70±2.39	<.0001
Basal E2	66.76±102.05	74.42±264.40	77.39±305.45	53.35±36.92	0.8418
Basal LH	4.13±1.75	4.08±1.71	3.82±1.78	4.04±1.75	0.3807
Basal T	0.86±3.81	0.72±3.58	0.57±1.95	0.43±0.38	0.7850
Duration of Infertility (Months)	66.02±46.90	65.96±46.92	64.52±44.95	70.23±55.54	0.9482
Dysmenorrhea					0.2178
Yes	254( 31.36%)	118( 30.10%)	30( 27.03%)	5( 16.13%)	.
No	556( 68.64%)	274( 69.90%)	81( 72.97%)	26( 83.87%)	.
Race					0.0034
Han	614( 75.80%)	328( 83.67%)	97( 87.39%)	29( 93.55%)	.
Vygur	85( 10.49%)	21( 5.36%)	5( 4.50%)	1( 3.23%)	.
Others	111( 13.70%)	43( 10.97%)	9( 8.11%)	1( 3.23%)	.

**Table 2:** Cycle outcomes of the treatments according to the number of embryos transferred to reach live birth (ETRL).

	From 1–3 ETRL (n=810)	From 4–6 ETRL (n=392)	From 7–9 ETRL (n=111)	More than 10 (n=31)	Overall *P *
GN dose	2887.62±1235.81	2707.54±1139.93	3017.82±748.73	2963.75±625.58	0.9482
(HCGDay)E2	3413.76±3499.26	5412.12±7646.49	4196.45±3757.99	4317.33±3867.31	0.0652
No. of retrieved oocytes	7.72±5.25	11.76±6.42	16.12±7.62	20.83±8.48	0.2450
No. of embryos transferred	2.02±0.71	4.68±0.79	7.77±0.73	11.35±1.50	0.0466
Pregnancy	335( 41.36%)	154( 39.29%)	50( 45.05%)	10( 32.26%)	
Miscarriage /pregnancy (%)	52( 15.52%)	28( 18.18%)	8( 16.00%)	4( 40.00%)	
Live birth	283( 34.94%)	126( 32.14%)	42( 37.84%)	6( 19.35%)	
Singleton (%)	248( 30.62%)	110( 28.06%)	39( 35.14%)	6( 19.35%)	
Twins (%)	35( 4.32%)	16( 4.08%)	3( 2.70%)		
No live birth	527( 65.06%)	266( 67.86%)	69( 62.16%)	25( 80.65%)	

## Data Availability

The data used to support the findings of this study are available from the corresponding author upon request.

## Supplementary Materials

Supplementary Materials The supplementary table provides number of patients in each number of ET.

## References

[1] ICMART (2018). *International Committee Monitoring Assisted Reproductive Technologies*.

[2] Fauser B. C. J. M., Devroey P., Macklon N. S. (2005). Multiple birth resulting from ovarian stimulation for subfertility treatment. *The Lancet*.

[3] Malizia B. A., Hacker M. R., Penzias A. S. (2009). Cumulative live-birth rates after in vitro fertilization. *The New England Journal of Medicine*.

[4] Nargund G., Waterstone J., Bland J. M. (2001). Cumulative conception and live birth rates in natural (unstimulated) IVF cycles. *Human Reproduction*.

[5] Cheng P., Duan T. (2016). China's new two-child policy: maternity care in the new multiparous era. *BJOG: An International Journal of Obstetrics & Gynaecology*.

[6] McLernon D. J., Steyerberg E. W., te Velde E. R., Lee A. J., Bhattacharya S. (2016). Predicting the chances of a live birth after one or more complete cycles of in vitro fertilisation: population based study of linked cycle data from 113 873 women. *BMJ*.

[7] Abuzeid M. I., Bolonduro O., La Chance J. (2014). Cumulative live birth rate and assisted reproduction: impact of female age and transfer day. *Facts, Views & Vision in Obgyn*.

[8] Drakopoulos P., Blockeel C., Stoop D. (2016). Conventional ovarian stimulation and single embryo transfer for IVF/ICSI. How many oocytes do we need to maximize cumulative live birth rates after utilization of all fresh and frozen embryos?. *Human Reproduction*.

[9] Briggs R., Kovacs G., MacLachlan V., Motteram C., Baker H. W. (2014). Can you ever collect too many oocytes?. *Human Reproduction*.

[10] Zhou J., Wang B., Hu Y., Sun H. (2017). Association between the number of oocytes retrieved and cumulative live birth rate in women aged 35–40 years undergoing long GnRH agonist IVF/ICSI cycles. *Archives of Gynecology and Obstetrics*.

[11] Devesa M., Tur R., Rodríguez I., Coroleu B., Martínez F., Polyzos N. P. (2018). Cumulative live birth rates and number of oocytes retrieved in women of advanced age. A single centre analysis including 4500 women ≥38 years old. *Human reproduction (Oxford, England)*.

[12] Soares S. R., Troncoso C., Bosch E. (2005). Age and uterine receptiveness: predicting the outcome of oocyte donation cycles. *The Journal of Clinical Endocrinology & Metabolism*.

[13] Pilar G., Carmen R. M., José D. L. S. (2003). The effect of pronuclear morphology on early development and chromosomal abnormalities in cleavage-stage embryos. *Human Reproduction*.

[14] Ludwig M., Finas D., Al-Hasani S., Diedrich K., Ortmann O. (1999). Oocyte quality and treatment outcome in intracytoplasmic sperm injection cycles of polycystic ovarian syndrome patients. *Human Reproduction*.

[15] Sahu B., Ozturk O., Ranierri M., Serhal P. (2008). Comparison of oocyte quality and intracytoplasmic sperm injection outcome in women with isolated polycystic ovaries or polycystic ovarian syndrome. *Archives of Gynecology and Obstetrics*.

[16] Report, ARTN, Assisted Reproductive Technology National Summary Report. Centers for Disease Control and Prevention. 2015

[17] Flenady V., Koopmans L., Middleton P. (2011). Major risk factors for stillbirth in high-income countries: a systematic review and meta-analysis. *The Lancet*.

[18] Morris J. K., De Vigan C., Mutton D. E., Alberman E. (2005). Risk of a Down syndrome live birth in women 45 years of age and older. *Prenatal Diagnosis*.

[19] Heffner L. J. (2004). Advanced maternal age — how old is too old?. *The New England Journal of Medicine*.

[20] Hamilton B. E., Martin J. A., Osterman M. J. (2015). Births: Final Data for 2014. *National Vital Statistics Reports*.

[21] Johnson J., Tough S., Wilson R. D. (2012). Delayed child-bearing. *Journal of Obstetrics and Gynaecology Canada*.

[22] Lim A. S., Tsakok M. F. (1997). Age-related decline in fertility: A link to degenerative oocytes?. *Fertility and Sterility*.

[23] Munne S., Alikani M., Tomkin G., Grifo J., Cohen J. (1995). Embryo morphology, developmental rates, and maternal age are correlated with chromosome abnormalities. *Fertility and Sterility*.

[24] Choi H. W., Park Y., Lee S., Lim C. K., Seo J. T., Yang K. M. (2016). Effects of maternal age on embryo quality and pregnancy outcomes using testicular sperm with intracytoplasmic sperm injection. *Clinical and Experimental Reproductive Medicine*.

[25] Scheffer J. B., Scheffer B. B., de Carvalho R. F., Rodrigues J., Grynberg M., Mendez Lozano D. H. (2017). Age as a predictor of embryo quality regardless of the quantitative ovarian response. *International Journal of Fertility & Sterility*.

[26] Twisk M., Mastenbroek S., van Wely M., Heineman M. J., Van der Veen F., Repping S. (2006). Preimplantation genetic screening for abnormal number of chromosomes (aneuploidies) in in vitro fertilisation or intracytoplasmic sperm injection. *Cochrane Database of Systematic Reviews*.

[27] Cheng E. Y., Hunt P. A., Naluai-Cecchini T. A. (2009). Meiotic recombination in human oocytes. *PLoS Genetics*.

[28] Eichenlaub-Ritter U., Wieczorek M., Lüke S., Seidel T. (2011). Age related changes in mitochondrial function and new approaches to study redox regulation in mammalian oocytes in response to age or maturation conditions. *Mitochondrion*.

[29] Pregnancy in Older Age Increases Stroke, Heart Attack Risk Years Later, 2016

[30] Jing X., Chen L. Z., Xue L. (2013). Meta-analysis of risk factors for childhood cerebral palsy during pregnancy. *Chinese Journal of Contemporary Pediatrics*.

[31] Fedorcsák P., Dale P. O., Storeng R. (2004). Impact of overweight and underweight on assisted reproduction treatment. *Human Reproduction*.

[32] Veleva Z., Tiitinen A., Vilska S. (2008). High and low BMI increase the risk of miscarriage after IVF/ICSI and FET. *Human Reproduction*.

[33] Robker R. L. (2008). Evidence that obesity alters the quality of oocytes and embryos. *Pathophysiology*.

[34] Bellver J., Ayllón Y., Ferrando M. (2010). Female obesity impairs in vitro fertilization outcome without affecting embryo quality. *Fertility and Sterility*.

[35] Rittenberg V., Seshadri S., Sunkara S. K., Sobaleva S., Oteng-Ntim E., El-Toukhy T. (2011). Effect of body mass index on IVF treatment outcome: an updated systematic review and meta-analysis. *Reproductive BioMedicine Online*.

[36] Lashen H., Ledger W., Bernal A. L. Extremes of body mass do not adversely affect the outcome of superovulation and in-vitro fertilization. *Human Reproduction*.

[37] Dechaud H., Anahory T., Reyftmann L., Loup V., Hamamah S., Hedon B. (2006). Obesity does not adversely affect results in patients who are undergoing in vitro fertilization and embryo transfer. *European Journal of Obstetrics & Gynecology and Reproductive Biology*.

[38] Maheshwari A., Stofberg L., Bhattacharya S. (2007). Effect of overweight and obesity on assisted reproductive technology—a systematic review. *Human Reproduction Update*.

[39] Schliep K. C., Mumford S. L., Ahrens K. A. (2015). Effect of male and female body mass index on pregnancy and live birth success after in vitro fertilization. *Fertility and Sterility*.

[40] Marquard K. L., Stephens S. M., Jungheim E. S. (2011). Polycystic ovary syndrome and maternal obesity affect oocyte size in in vitro fertilization/intracytoplasmic sperm injection cycles. *Fertility and Sterility*.

[41] Machtinger R., Combelles C. M., Missmer S. A., Correia K. F., Fox J. H., Racowsky C. (2012). The association between severe obesity and characteristics of failed fertilized oocytes. *Human Reproduction*.

[42] Best D., Avenell A., Bhattacharya S. (2017). How effective are weight-loss interventions for improving fertility in women and men who are overweight or obese? A systematic review and meta-analysis of the evidence. *Human Reproduction Update*.

