# Supplementary material for: Use of Cumulative Live Birth Rate per Total Number of Embryos to Calculate the Success of IVF in Consecutive IVF Cycles in Women Aged ≥35 Years
Source: Biomed Res Int. 2019 Jun 26;2019:6159793. doi: 10.1155/2019/6159793 (PMC6617928; doi:10.1155/2019/6159793)
Supplement: Supplementary Materials — The supplementary table provides number of patients in each number of ET. [file 6159793.f1.pdf]

Supplementary table1

No. of embryo transferred	Begin total patient	Group patient	No. of live birth	No. of failure	Cumulative probability	Standard Error
1	1344	195	47	148	0.0350	0.005
2	1149	404	172	232	0.1794	0.011
3	745	211	64	147	0.2499	0.0131
4	534	203	67	136	0.3440	0.0157
5	331	111	34	77	0.4114	0.0179
6	220	78	25	53	0.4783	0.0202
7	142	45	15	30	0.5334	0.0225
8	97	46	19	27	0.6248	0.0261
9	51	20	8	12	0.6837	0.0292
10	31	13	2	11	0.7041	0.0306
11	18	6	1	5	0.7205	0.0331
12	12	4	1	3	0.7438	0.0376
13	8	5	1	4	0.7758	0.0445
14	3	2	1	1	0.8505	0.0678
15	1	1	0	1	0.8505	0.0678
